# A Novel Intergenic Gene Between *SLC8A1* and *PKDCC*-*ALK* Fusion Responds to *ALK* TKI WX-0593 in Lung Adenocarcinoma: A Case Report

**DOI:** 10.3389/fonc.2022.898954

**Published:** 2022-06-30

**Authors:** Jia Du, Baoming Wang, Mengxia Li, Chunyang Wang, Tonghui Ma, Jinlu Shan

**Affiliations:** ^1^ Cancer Center, Daping Hospital, Army Medical University, Chongqing, China; ^2^ Department of Translational Medicine, Genetron Health (Beijing), Co. Ltd., Beijing, China

**Keywords:** fusion, *intergenic-ALK*, inhibitor, WX-0593, *SLC8A1*, *PKDCC*

## Abstract

**Background:**

Expanding the druggable novel anaplastic lymphoma kinase (*ALK*) fusions list is crucial to the precise treatment of patients with cancer with positive *ALK* fusions. The *intergenic-ALK* fusions accounted for a substantial proportion of *ALK* fusions. However, they were typically considered of limited clinical significance due to the obscure functional partners. In this case report, a patient carrying *intergenic-ALK* fusion presents an excellent outcome after taking the new second-generation tyrosine kinase inhibitor (TKI) candidate, WX-0593.

**Case Presentation:**

A 47-year-old Chinese female patient diagnosed with IVB lung adenocarcinoma was admitted to the hospital with large dimension lesions in the left lobe of the lung. After 1 week of first line chemotherapy, no response was found. A novel *ALK* rearrangement generated by a fusion of the intergenic region between *SLC8A1* and *PKDCC* to the intron 19 of *ALK* was presented after next-generation sequencing and was further confirmed by Sanger’s sequencing. High expression of *ALK* was revealed by immunohistochemistry. The patient was directed to engage in phase III clinical trial (NCT04632758) and received an orally active second-generation *ALK* inhibitor WX-0593. Over the course of 17 months, the partial response was obtained without significant side effects.

**Conclusion:**

In summary, a patient with non–small cell lung cancer harboring a novel *intergenic-ALK* fusion, whose intergenic breakpoint was located between *SLC8A1* and *PKDCC*, benefited from a potent *ALK* TKI candidate WX-0593. This finding extended the scope of targetable *ALK* fusions. More importantly, it highlighted the advantages of next-generation sequencing in identifying rare but functional *ALK* fusions, which eventually benefit patients.

## Introduction

Anaplastic lymphoma kinase (*ALK*) gene fusions drive genetic alterations and critical molecular targets in around 3%~5% of non–small cell lung cancer (NSCLC) (1). For the treatment of patients with *ALK* rearrangement-positive NSCLC, the first-generation *ALK* tyrosine kinase inhibitor (TKI) crizotinib (2), second-generation [ceritinib (3), alectinib (4), and brigatinib (5)], and third-generation [lorlatinib (6)] *ALK* TKIs have been approved as an effective treatment. Because of their rarity, newly confirmed *ALK* fusions, in addition to the conventional *EML4-ALK* fusion, represent significant difficulties in targeted *ALK* TKI therapy in the clinic. Clinical outcomes vary according to fusion partners and specific TKIs (7). Therefore, an accurate diagnosis of functional *ALK* fusions is crucial for successful NSCLC treatment. In contrast to the typical form of *ALK* fusions, the rare *intergenic*-*ALK* fusions, whose breakpoint localized in the intergenic regions, are theoretically to be unfunctional due to the missing chimeric full coding transcripts. Here, for the first time, we reported a novel *intergenic*-*ALK* fusion, whose intergenic region was between *SLC8A1* and *PKDCC* and fused with the exon 20 of ALK in a patient with NSCLC. The patient achieved a long-term therapeutic benefit after receiving the potent second-generation candidate *ALK* TKI WX-0593 treatment.

## Case Presentation

**Figure 1:**
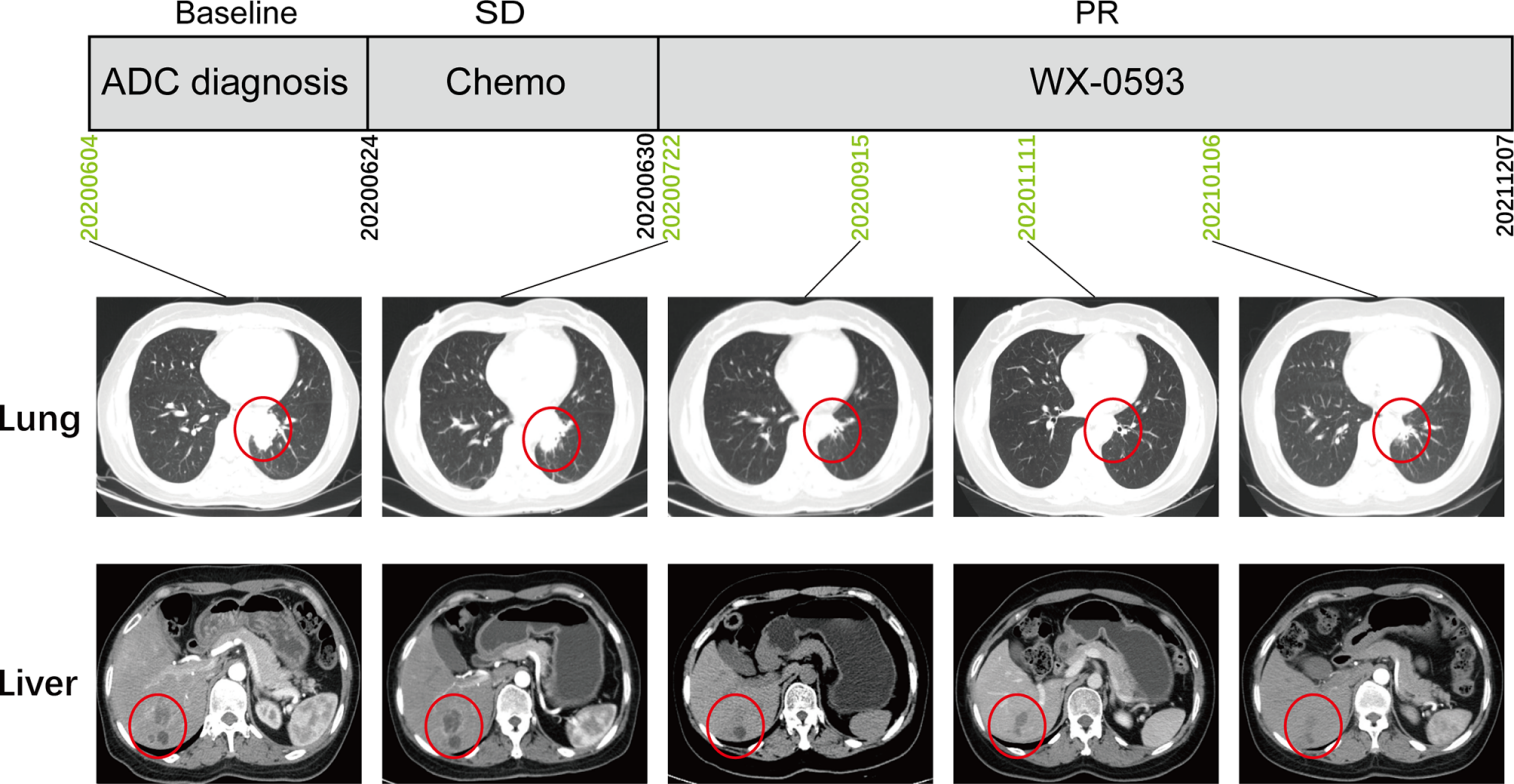
The treatment scheme and representative CT scan images during treatment. ADC, adenocarcinoma; SD, stable disease; PR, partial response.

This case report was approved by the Ethics Committee of Daping Hospital [2022(03)]. A 47-year-old Chinese female patient was admitted to the hospital with paroxysmal abdominal pain in June 2020. Computed tomography (CT) scans revealed a space-occupying lesion in the left lung lobe, as well as multiple masses in the liver and skeleton. IVB lung adenocarcinoma (cT2N1M1c) was diagnosed on the basis of the pathological results from tissue biopsy and CT. Lung tumor biopsies were submitted to Genetron Health Inc. (Beijing, China) for Next Generation Sequencing (NGS) with an 825 cancer-related gene DNA panel (Onco Panscan™) for comprehensive molecular profiling. During the DNA-NGS analysis, the patient received 1 week of chemotherapy with pemetrexed disodium (0.8 g) and nedaplatin (100 mg) and did not show any response (**Figure 1**).

**Figure 2:**
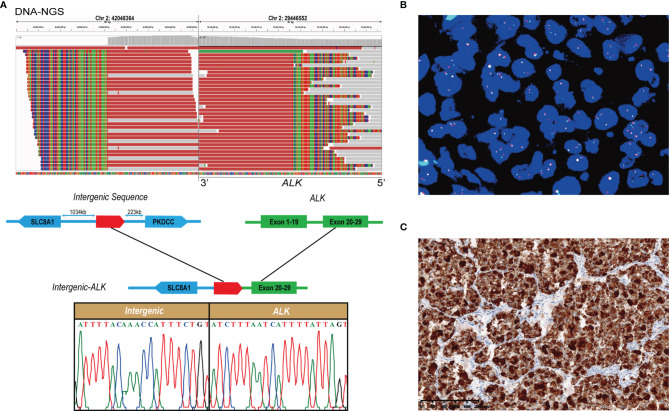
Identification of a novel intergenic ALK fusion. **(A)** Sequence analysis of the *intergenic-ALK* fusion. Upper panel showed reads of fusion on Integrative Genomic Viewer. Lower panel showed a schematic presentation of breakpoints and the Sanger’s sequencing result. **(B)** FISH analysis showed fused yellow signals (negative signal), single green signals (positive signal), and single red signals (positive signal) in the patient’s specimen. **(C)** Immunohistochemical staining with anti-ALK antibody (D5F3) revealed a high level of ALK protein.

The DNA sequencing identified a novel *ALK* fusion with a mutation frequency of 5.3% (**Figure 2A**). Integrative Genomics Viewer revealed a novel *intergenic*-*ALK* rearrangement generated by a fusion of the intergenic region between *SLC8A1* and *PKDCC* to the intron 19 of *ALK*, and Sanger’s sequencing result supported the accuracy of DNA-NGS detection. Furthermore, the gene fusion was confirmed by fluorescence *in situ* hybridization (FISH) with *ALK* break-apart probe (Healthcare, NMPA: 20183400004) (**Figure 2B**). A high level of *ALK* protein expression was further validated with immunohistochemistry (Ventana ALK (D5F3^®^) XP^®^, #3633, Cell Signaling Technology) (**Figure 2C**).

The patient was referred to enroll in phase III clinical trial (NCT04632758) and was randomly administered an orally active second-generation *ALK* inhibitor WX-0593. An initial dose of 60 mg was administered for 1 week, followed by a maintenance dose of 180 mg. According to the Response Evaluation Criteria for Solid Tumors 1.1 guidelines, the CT scan indicated a partial response (PR) lasting longer than 22 months, with no noticeable side effects (**Figure 1**).

## Discussion

With the development of NGS technology, more than 90 *ALK* fusion partners have been discovered to date (8). It is generally believed that the functional protein of the partner gene at the N-terminus of *ALK* could result in continuous *ALK* gene activation driven by the promoter of the partner gene. Theoretically, the rare type of *intergenic-ALK* fusion, whose partner lacked a promoter, will fail to respond to TKIs. Sporadic cases showed that some *intergenic-ALK* fusions were sensitive to the *ALK* TKIs (8). However, it has not been fully identified due to a lack of knowledge concerning the precise mechanism (9). Therefore, it is vital to expand the list of targetable intergenic fusions.

To date, three generations of *ALK* TKIs, including but not limited to crizotinib, alectinib, and lorlatinib, have been developed. These inhibitors present an exceptional capacity to lengthen the lives of patients with *ALK* fusions. Despite the excellent results of these TKIs, newly developed local *ALK* TKIs were required to reduce the financial burden on the patients and national health insurance. *In vitro* and *in vivo* preclinical models revealed that WX-0593, a potent orally active second-generation *ALK* and ROS1 inhibitor, showed robust antitumor activity. Furthermore, it presented the noticeable safety and efficacy in patients with *ALK*-positive and *ROS1*-positive NSCLC in phase I clinical trial (NCT03389815) (10).

In this study, the patient with novel *intergenic-ALK* (chr2_42048364; A20) fusion showed an excellent prognosis outcome after being treated with WX-0593. The soft tissue masses in the lung and liver diminished dramatically, which had obtained a PR after 17 months of therapy. The patient continues to receive WX-0593 medication while preparing this manuscript. Positive therapeutic outcomes in our investigation demonstrated that *intergenic-ALK* fusion could be considered as a potential oncogenic mutation by stimulating the overexpression of *ALK* proteins. We infer that the intergenic region at the 5’ end functions as a strong promoter in this unique fusion variant. Nonetheless, the oncogenic and molecular processes of this fusion will need to be further investigated.

Despite the great novelty of our results, several limitations still need to be discussed. RNA-based NGS is preferred to DNA-based NGS for fusion detection based on the NCCN Guidelines Version 1.2022 for NSCLC. However, because of the limited number of retained specimens, the RNA-NGS was not performed. It could not be precisely annotated whether the newly discovered fusion forms differed at the DNA and RNA levels. To confirm the biological function of the *intergenic-ALK* fusion in cancer, additional research is required.

## Conclusion

In summary, an *ALK*-TKI candidate, WX-0593, was effectively treated a patient with NSCLC with novel *intergenic-ALK* fusion. This case broadened the breadth of *ALK* fusions that can be targeted and highlighted the utility of NGS in mining rare but functional *ALK* fusions, which eventually bring benefits to the patients.

## Data Availability Statement

The original contributions presented in the study are included in the article/supplementary materials. Further inquiries can be directed to the corresponding author.

## Ethics Statement

This case report was approved by the Ethics Committee of Daping Hospital [2022(03)]. The patients/participants provided their written informed consent to participate in this study. Written informed consent was obtained from the individual(s) for the publication of any potentially identifiable images or data included in this article.

## Author Contributions

JD and BW contributed to the experiment performing and manuscript writing. CW and TM participated to the data analysis. ML provided the clinical samples and relevant information. JS designed and optimized the experiment. All authors contributed to the article and approved the submitted version.

## Funding

Clinical medical technology innovation ability training program (2019CXLCB002)

## Conflict of Interest

Authors BW, CW and TM were employed by Genetron Health (Beijing), Co. Ltd.

The remaning authors declare that the research was conducted in the absence of any commercial or financial relationships that could be construed as a potential conflict of interest.

## Publisher’s Note

All claims expressed in this article are solely those of the authors and do not necessarily represent those of their affiliated organizations, or those of the publisher, the editors and the reviewers. Any product that may be evaluated in this article, or claim that may be made by its manufacturer, is not guaranteed or endorsed by the publisher.

## References

[1] Du X Shao Y Qin HF Tai YH Gao HJ . ALK-Rearrangement in Non-Small-Cell Lung Cancer (NSCLC). Thorac Cancer (2018) 9:423–30. 10.1111/1759-7714.12613 PMC5879058 29488330

[2] Shaw AT Kim D-W Nakagawa K Seto T Crinó L Ahn M-J . Crizotinib Versus Chemotherapy in Advanced ALK-Positive Lung Cancer. N Engl J Med (2013) 368:2385–94. 10.1056/NEJMoa1214886 23724913

[3] Shaw AT Kim D-W Mehra R Tan DS Felip E Chow LQ . Ceritinib in ALK-Rearranged Non–Small-Cell Lung Cancer. N Engl J Med (2014) 370:1189–97. 10.1056/NEJMoa1311107 PMC4079055 24670165

[4] Peters S Camidge DR Shaw AT. Gadgeel S Ahn JS Kim D-W . Alectinib Versus Crizotinib in Untreated ALK-Positive Non–Small-Cell Lung Cancer. N Engl J Med (2017) 377:829–38. 10.1056/NEJMoa1704795 28586279

[5] Camidge DR Kim HR Ahn M-J Yang JC-H Han J-Y Lee J-S . Brigatinib Versus Crizotinib in ALK-Positive non–Small-Cell Lung Cancer. N Engl J Med (2018) 379:2027–39. 10.1056/NEJMoa1810171 30280657

[6] Solomon BJ Besse B Bauer TM Felip E Soo RA Camidge DR . Lorlatinib in Patients With ALK-Positive Non-Small-Cell Lung Cancer: Results From a Global Phase 2 Study. Lancet Oncol (2018) 19:1654–67. 10.1016/S1470-2045(18)30649-1 30413378

[7] Childress MA Himmelberg SM Chen H Deng W Davies MA Lovly C. M. . ALK Fusion Partners Impact Response to ALK Inhibition: Differential Effects on Sensitivity, Cellular Phenotypes, and Biochemical Properties. Mol Cancer Res MCR (2018) 16:1724–36. doi: 10.1158/1541-7786.Mcr-18-0171 PMC6214753 30002191

[8] Ou S-HI Zhu VW Nagasaka M . Catalog of 5’fusion Partners in ALK-Positive NSCLC Circa 2020. JTO Clin Res Rep (2020) 1:100015. 34589917 10.1016/j.jtocrr.2020.100015 PMC8474466

[9] Li W Liu Y Li W Chen L Ying J . Intergenic Breakpoints Identified by DNA Sequencing Confound Targetable Kinase Fusion Detection in NSCLC. J Thorac Oncol Off Publ Int Assoc Study Lung Cancer (2020) 15:1223–31. doi: 10.1016/j.jtho.2020.02.023 32151779

[10] Shi Y-K Fang J Zhang S Liu Y Wang L Si M . Safety and Efficacy of WX-0593 in ALK-Positive or ROS1-Positive Non-Small Cell Lung Cancer. Ann Oncol (2019) 30:v607–8.

